# Clinical and Oculomotor Correlates With Freezing of Gait in a Chinese Cohort of Parkinson’s Disease Patients

**DOI:** 10.3389/fnagi.2020.00237

**Published:** 2020-07-31

**Authors:** Li Wu, Qin Wang, Lei Zhao, Chun-Yan Jiang, Qian Xu, Si-Cheng Wu, You-Rong Dong, Qing He, Wei Chen, Jian-Ren Liu

**Affiliations:** ^1^Department of Neurology, Shanghai Ninth People’s Hospital, Shanghai Jiao Tong University School of Medicine, Shanghai, China; ^2^Department of Neurology, Zhongshan Hospital Fudan University, Shanghai, China; ^3^Biostatistics Office of Clinical Research Center, Shanghai Ninth People’s Hospital, Shanghai Jiao Tong University School of Medicine, Shanghai, China

**Keywords:** Parkinson’s disease, freezing of gait, videonystagmography, saccade latency, non-motor symptom

## Abstract

Accumulating evidence suggests that freezing of gait (FOG) is a unique gait disturbance in Parkinson’s disease (PD), and its pathophysiology is not fully elucidated. The present study aims to investigate the clinical and oculomotor associations with FOG in Chinese PD patients. From Jan 2017 to Dec 2019, a total of 210 PD patients were consecutively registered for FOG evaluation based on item-3 of the Freezing of Gait Questionnaire (FOGQ). We explored the demographic, motor, and non-motor symptom differences in FOG positive (PD+FOG, *n* = 45) vs. negative (PD-FOG, *n* = 165) group. In addition, 40 PD patients and 37 healthy controls (HC) also underwent oculomotor test via videonystagmography (VNG). Visually guided saccade (VGS) latency, saccade accuracy and gain in smooth pursuit eye movement (SPEM) at three frequencies of horizontal axis were compared among PD+FOG (*n* = 20), PD-FOG (*n* = 20), and HC (*n* = 37). Compared with PD-FOG, PD+FOG had longer disease duration, more severe motor symptoms, lower cognitive scores, more severe depressive and autonomic impairments, as well as higher daily levodopa equivalent dosage. FOG occurred more frequently in patients with wearing-off. VNG subgroup analysis demonstrated that PD+FOG had prolonged saccade latency and decreased saccade accuracy relative to PD-FOG or HC. SPEM gain at 0.1 and 0.2 Hz was also decreased in PD+FOG compared with HC. Furthermore, prolonged saccade latency was correlated with higher FOGQ scores in PD patients. Our results verify that PD with FOG patients suffer from more severe motor and non-motor symptoms, indicating more extensive neurodegeneration. Prolonged saccade latency could be a practical oculomotor parameter both for identification and progression of FOG in PD.

## Introduction

Freezing of gait (FOG) is defined as a brief, episodic absence or marked reduction of forward progression of the feet despite having the intention to walk. It usually occurs relatively in late stage of Parkinson’s Disease (PD) and increases the risk of falls, contributing to impaired quality of life (Nieuwboer and Giladi, 2013). FOG in PD has been reported to be associated with several clinical features such as more severe motor symptoms, lower limb onset, cognitive impairment, and higher daily dose of levodopa (Giladi et al., 2001; Perez-Lloret et al., 2014; Amboni et al., 2015). However, the detailed motor and non-motor clinical correlates are not well investigated in China, and the potential pathophysiology is not fully understood yet. We currently lack biomarkers to predict this subset.

Accumulating evidence since 1983 showed that PD patients also had eye movement abnormalities including saccadic and smooth pursuit eye movement (SPEM) system (White et al., 1983) and the increased inhibition to superior colliculus (SC) activity from basal ganglia output may be the cause (Anderson and MacAskill, 2013). However, the clinical implication of these oculomotor impairments is less investigated in PD. Oculomotor impairment may influence balance and posture/gait control of a subject. It is reported that PD with postural instability had increased anti-saccade latency (Ewenczyk et al., 2017); and PD with FOG had reduced postural control (Schlenstedt et al., 2016). We hypothesized that PD with FOG may have more obvious oculomotor deficits.

Therefore, the present study firstly investigated the motor and non-motor symptom correlations with FOG by detailed scales in Shanghai, China; In addition, we tried to explore the oculomotor associations with FOG by videonystagmography (VNG), aiming to find biomarkers for this unique subset of PD.

## Materials and Methods

### Subjects

From Jan 2017 to Dec 2019, a total of 210 PD patients, who met the Movement Disorder Society (MDS) clinical diagnostic criteria (Postuma et al., 2015) were consecutively recruited from the movement disorders clinic (Department of Neurology, Shanghai Ninth People’s Hospital, Shanghai Jiao Tong University School of Medicine, Shanghai, China). None of the patients had undergone functional neurosurgery for PD. We excluded individuals with other chronic or acute brain diseases. 37 healthy controls were recruited from the community nearby. The age of all the participants ranged from 50 to 80 years old. All patients and controls underwent the Chinese equivalent of the Mini Mental State Examination (Katzman et al., 1988). We excluded those whose MMSE score fell below 20 for individuals that received 6 years of schooling or less (primary school education) and below 24 for individuals that received more than 6 years of schooling (junior high and above). Written informed consents were obtained from all participants. The study was approved by the Ethics Committee of Shanghai Ninth People’s Hospital, Shanghai Jiao Tong University School of Medicine.

### Clinical Profiles

Demographic and clinical characteristics of PD were listed in Table 1. For these patients, motor severity was measured with modified Hoehn and Yahr (H&Y) stage (Hoehn and Yahr, 1967), and Unified Parkinson’s Disease Rating Scale part III (UPDRS-III; Richards et al., 1994) performed in the “on” state. Tremor, akinetic/rigid, postural, and gait instability scores were further calculated according to the report from Kang et al. (2005). UPDRS-II was used to evaluate activities of daily living. FOG was considered present, when subjects had a score of >0 on item 3 of Freezing of Gait questionnaire (FOG-Q; Giladi et al., 2000). The total burden of non-motor symptoms was measured with Non-Motor Symptoms Questionnaire (NMSQuest; Richards et al., 1994). Olfactory function was assessed by SS-16 (Burghart Messtechnik, Wedel, Germany) as our previous report (Chen et al., 2012). REM Behavior Disorder Screening Questionnaire (RBDSQ) was used to screen clinical possible RBD (cpRBD; Nomura et al., 2011). The severity of depressive symptom was assessed by 17-item Hamilton Rating Scale for Depression (HAMD-17; Hamilton, 1960). The Scales for Outcomes in PD Autonomic Dysfunction (SCOPA-AUT) was used as a measurement of autonomic disfunction (Verbaan et al., 2007). Total cognitive function was assessed by MMSE (Katzman et al., 1988) and Montreal Cognitive Assessment Basic (MoCA-BC; Chen et al., 2016). Daily L-dopa equivalent dosage (LED) was calculated as reported (Tomlinson et al., 2010).

**TABLE 1:** Demographic and clinical features in Parkinson’s disease with FOG.

	PD with FOG	PD without FOG	*P-*value
Number, *n*	45	165	
Age, *y*	66.5 ± 8.9	66.2 ± 8.9	0.376
M/F, *n*	29/16	94/71	0.367
Disease duration, y	4.0 (1.5, 6.0)	2.0 (1.0, 3.0)	0.009**
Hoehn and Yahr stage			<0.001***
1–2, *n* (%)	25 (55.6)	150 (90.9)	
>2, *n* (%)	20 (44.4)	15 (9.1)	
UPDRS-II (0–52)	14.0 (10.0, 18.0)	7.0 (4.0, 10.0)	<0.001***
UPDRS-III (0–108)	27.5 (18.0, 43.0)	15.5 (9.0, 24.0)	<0.001***
Tremor score	4.0 (0.0, 7.0)	2.0 (1.0, 4.0)	0.022*
Bradykinesia and rigidity score	15.0 (10.0, 27.0)	10.0 (6.0, 18.0)	<0.001***
Posture and gait score	4.0 (3.0, 5.0)	2.0 (1.0,3.0)	<0.001***
Motor fluctuation			
Wearing-off, *n* (%)	19 (42.2)	19 (11.5)	<0.001***
Dyskinesia, *n* (%)	2 (4.4)	3 (1.8)	0.291
NMSQuest (0–30)	9.9 ± 4.5	6.8 ± 4.1	<0.001***
SS-16 (0–16)	7.5 (4.0, 9.0)	8.0 (6.0, 10.0)	0.129
RBDSQ (0–13)	3.5 (1.0, 7.0)	3.0 (1.0, 6.0)	0.685
HAMD-17 (0–17)	7 (3.0, 12.0)	4 (1.5, 8.5)	0.022*
SCOPA-AUT (0–63)	13.5 (8.0, 17.0)	9.0 (6.0, 12.0)	<0.001***
MMSE (0–30)	27.4 ± 1.9	27.65 ± 2.5	0.532
MoCA-BC (0–30)	20.9 ± 5.1	22.5 ± 4.3	0.038*
LED, mg/d	400.0 (150.0, 600.0)	150.0 (0, 375.0)	<0.001***

FOG, freezing of gait; M/F, male/female; UPDRS, Unified Parkinson’s Disease Rating Scale; NMSQuest, Non-motor Symptoms Questionnaire; SS-16, 16-item odor identification test from Sniffin’ Sticks; RBDSQ, REM Behavior Disorder Screening Questionnaire; HAMD-17, 17-item Hamilton Rating Scale for Depression; SCOPA-AUT, The Scale for Outcomes in PD autonomic dysfunction; MMSE, Mini Mental State Examination; MoCA-BC, Montreal Cognitive Assessment Basic; LED, L-dopa equivalent dosage. *p < 0.05; **p < 0.01; and ***p < 0.001.

### Videonystagmography Evaluation

To minimize the influence of involuntary movements, subjects with head tremor or dyskinesia were excluded for oculomotor evaluation. Finally, 40 out of 210 PD patients and 37 healthy controls underwent oculomotor test by a VisualEyes 4 channel VNG (Micromedical Technologies), which acquired binocular movement samples at 120 Hz. Subjects remained seated in darkness for 2 min before testing. Horizontal VGS and SPEM at three frequencies (0.1, 0.2, and 0.4 Hz) were performed in all participants. We recorded these oculomotor parameters: saccade latency, saccade accuracy, and gain of SPEM at three different frequencies separately.

### Statistical Analysis

Statistical analyses were performed utilizing SPSS (version 23.0 for Windows) and MedCalc (version 18.0 for Windows). GraphPad Prism (version 5.0 for Windows) was used for plotting the graphics. Shapiro–Wilk test was used to determine whether sample data is normally distributed. We used mean and standard deviation (SD) for numerical variables (age, etc.) with normal distribution; median and interquartile range (IQR, 25th–75th percentile) for those with skewed distributions. To compare categorical data among groups, we applied the chi-square test or Fisher’s exact test. We analyzed the continuous variables by in-dependent *t* test, one-way analysis of variance (ANOVA) or non-parametric Kruskal–Wallis test, depending on whether the data were normally distributed or not. The correlation between oculomotor dynamics and clinical parameters was analyzed by Spearman rank correlation. A 95% confidence interval was calculated for the correlation coefficient. Binary logistic regression analysis by forward stepwise was used to determine the independent associated factors of FOG in PD patients. The input variables were those with significant difference in univariate analyses. Odds ratios (ORs) and 95% confidence intervals (CIs) were reported accordingly. In all analyses, a two-tailed *p*-value < 0.05 was considered statistically significant.

## Results

### Demographic and Clinical Features of PD Patients With FOG

The age at examination of the patients was 65.49 ± 8.88 years, and 123 (58.57%) of whom were males. The disease duration was 3.06 ± 3.34 years. Thirty-seven (17.62%) of them reported family history. One hundred and seventy-five (83.33%) patients had H&Y stage 1–2; whereas 35 (16.67%) patients had H&Y stage >2. Thirty-eight (18.10%) patients had wearing-off and 5 (2.38%) had dyskinesia.

There were 45 patients with FOG positive (PD+FOG), occupying 21.4% of all enrolled PD. Compared with PD-FOG, PD+FOG had longer disease duration (*p* = 0.009), and more severe motor symptoms as revealed by UPDRS-III score (*p* < 0.001), and H&Y stage (*p* < 0.001); In addition, PD with FOG had more non-motor symptom burden as shown with higher score of NMSQuest (*p* < 0.001), HAMD-17 (*p* = 0.022), SCOPA-AUT (*p* < 0.001), and lower score of MoCA-BC (*p* = 0.038). Regarding medications, PD with FOG had more daily LED (*p* < 0.001; Table 1).

Binary logistic regression analysis showed that UPDRS-III (OR = 1.052; 95%CI: 1.021–1.085; *p* = 0.001), NMSquest (OR = 1.124; 95%CI:1.025–1.232; *p* = 0.013) and wearing- off (OR = 3.383; 95%CI:1.454–7.871; *p* = 0.005) were independent associated factors for FOG (Table 2).

**TABLE 2:** Binary logistic regression analysis for independent associated factors with FOG in PD.

Items	B	*P*	OR	95%CI for OR
UPDRS-III	0.051	0.001**	1.052	1.021–1.085
NMSquest	0.117	0.013*	1.124	1.025–1.232
Wearing-off	1.219	0.005**	3.383	1.454–7.871

FOG, freezing of gait; UPDRS, Unified Parkinson’s Disease Rating Scale; NMSQuest, Non-motor Symptoms Questionnaire; OR, odds ratio; CI, confidential interval; *p < 0.05; and **p < 0.01.

### Oculomotor Performances in PD Patients With FOG

Forty PD patients underwent oculomotor evaluation, the current age of whom at examination was 66.35 ± 5.07 years, and 57.50% (*n* = 23) were males. The disease duration was 4.75 ± 3.16 years. Twenty-six (65.00%) patients had H&Y stage 1–2; whereas 14 (35.00%) patients had H&Y stage >2. Seventeen (42.50%) patients had wearing-off (Supplementary Table 1). There were no significant differences in age, gender distribution or MMSE score among the three groups: PD+FOG, PD-FOG, and HC (Supplementary Table 1). Also, the disease duration, H&Y stage and UPDRS-III was comparable between PD with and without FOG (Supplementary Table 1).

**FIGURE 1:**
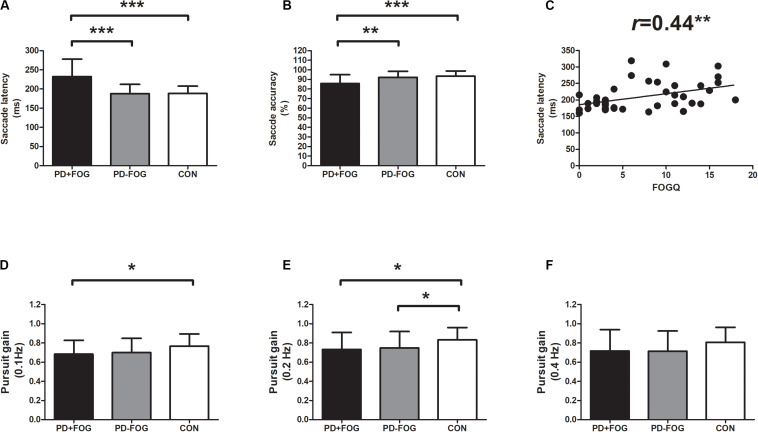
Oculomotor parameters in PD with FOG. **(A)** Saccade latency was increased in PD+FOG group, compared with PD-FOG or healthy control group; **(B)** Saccade accuracy was decreased both in PD+FOG or PD-FOG group, compared with healthy control group; **(C)** saccade latency was positively associated with FOGQ score in PD; **(D,E)** PD had decreased gain of smooth pursuit eye movement at 0.1 Hz **(D)** and 0.2 Hz **(E)** relative to healthy controls; **(F)** PD+FOG group had marginally decreased gain of smooth pursuit eye movement at 0.4 Hz, relative to healthy control group (*p* = 0.095). Notes: PD+FOG, Parkinson’s disease with freezing of gait; PD-FOG, Parkinson’s disease without freezing of gait; CON, healthy controls. Error bar represents standard deviation. *p < 0.05; **p < 0.01; and ***p < 0.001.

Oculomotor analysis revealed that saccade latency was not associated with other oculomotor dynamics such as saccade accuracy or SPEM gain. PD+FOG group had prolonged saccade latency (both *p* < 0.001) and decreased saccade accuracy (*p* = 0.003, *p* < 0.001, respectively) relative to PD-FOG or HC group (Table 3 and Figures 1A,B). Meanwhile, SPEM gain at 0.1 Hz (*p* = 0.03), and 0.2 Hz (*p* = 0.02) was decreased in PD+FOG compared with HC group (Table 3 and Figures 1D,E). PD-FOG also had decreased SPEM gain at 0.2 Hz (*p* = 0.045; Table 3 and Figure 1E) in comparison with HC. A marginal trend was noted for SPEM gain at 0.4 Hz (Table 3 and Figure 1F). There was a moderate positive correlation (*r* = 0.44, *p* = 0.004) between saccade latency and FOGQ score in PD patients (Table 4 and Figure 1C).

**TABLE 3:** Oculomotor characteristics in Parkinson’s disease with FOG.

Items	PD with FOG	PD without FOG	Healthy controls	*P-value*
	Group A	Group B	Group C	A vs. C; B vs. C; A vs. B
Number	20	20	37	
Saccade latency, ms	232.50 ± 45.54	188.17 ± 23.97	188.43 ± 18.87	<0.001***; 0.975; <0.001***
Saccade accuracy (0–100%)	85.77 ± 9.20	92.20 ± 6.24	93.57 ± 5.25	<0.001***; 0.466; 0.003**
Smooth pursuit gain (0.1 Hz; 0–1)	0.68 ± 0.14	0.70 ± 0.15	0.77 ± 0.13	0.030*; 0.076; 0.722
Smooth pursuit gain (0.2 Hz; 0–1)	0.73 ± 0.18	0.75 ± 0.17	0.83 ± 0.13	0.020*; 0.045*; 0.765
Smooth pursuit gain (0.4 Hz; 0–1)	0.72 ± 0.22	0.71 ± 0.21	0.81 ± 0.16	0.095; 0.084; 0.960

FOG, freezing of gait; *p < 0.05; **p < 0.01; and ***p < 0.001.

**TABLE 4:** The correlation between key oculomotor dynamics and clinical phenotypes.

	Saccade latency			Pursuit gain at 0.2 Hz		
	*p*	*r*	95% CI	*p*	*r*	95% CI
Age	0.391	0.139	−0.180–0.432	0.255	−0.184	−0.469–0.135
Gender	0.403	−0.136	−0.429–0.183	0.957	0.009	−0.319–0.304
Disease duration	0.757	0.051	−0.265–0.356	0.188	−0.212	−0.491–0.106
UPDRS-III	0.270	0.179	−0.141–0.464	0.220	−0.198	−0.480–0.121
Wearing-off	0.484	0.114	−0.205–0.411	0.366	−0.147	−0.438–0.172
FOG	0.0004***	0.533	0.266–0.724	0.700	−0.063	−0.367–0.254
FOGQ	0.004***	0.443	0.153–0.663	0.181	−0.216	−0.494–0.102
SS-16	0.539	−0.100	−0.399–0.218	0.653	−0.073	−0.376–0.244
RBDSQ	0.276	0.176	−0.143–0.462	0.989	−0.002	−0.314–0.310
HAMA-17	0.011**	0.398	0.099–0.631	0.390	−0.140	−0.432–0.180
SCOPA-AUT	0.055	0.305	−0.007–0.563	0.824	0.036	−0.278–0.344
MMSE	0.792	00.043	−0.350–0.272	0.758	0.050	−0.265–0.356
MoCA-BC	0.116	−0.253	−0.523–0.064	0.899	−0.021	−0.330–0.293
LED	0.514	0.108	−0.215–0.409	0.031*	−0.346	−0.596–0.034

CI, confidence interval; UPDRS-III, Unified Parkinson’s Disease Rating Scale part III; FOG, freezing of gait; FOGQ, freezing of gait questionnaire; SS-16, 16-item odor identification test from Sniffin’ Sticks; RBDSQ, REM Behavior Disorder Screening Questionnaire; HAMD-17, 17-item Hamilton Rating Scale for Depression; SCOPA-AUT, The Scale for Outcomes in PD autonomic dysfunction; MMSE, Mini Mental State Examination; MoCA-BC, Montreal Cognitive Assessment Basic; LED, L-dopa equivalent dosage. Bolded values are variables with statistical significance; *p < 0.05; **p < 0.01; and ***p < 0.001.

To further investigate the correlation between oculomotor dynamics and clinical parameters in PD, Spearman rank correlation tests were employed, We found that saccade latency was closely related to FOG status and FOGQ, In addition, it was also significantly correlated with HAMA-17 score (*r* = 0.398, *p* = 0.011; Table 4); SPEM gain at 0.2 Hz was negatively associated with LED (*r* = −0.346, *p* = 0.031; Table 4). However, saccade latency, as well as SPEM gain, was not correlated with other clinical parameter such as age, disease duration, motor, or cognitive impairments in PD.

## Discussion

This is a cross-sectional study with both clinical and ocular movement evaluation from a cohort of PD patients in Shanghai, China. Based on 210 PD patients with detailed phenotype analysis, we verified that PD+FOG had more severe motor and non-motor symptom burden in comparison with PD-FOG; VNG subgroup analysis found that PD+FOG had prolonged saccade latency relative to PD-FOG and HC, and delayed saccade latency was correlated with higher FOGQ scores in PD patients.

Given that PD with freezers had longer disease duration, more severe motor symptom and more frequency of wearing-off in our results, confirming that striatal dopaminergic denervation is critical in the pathophysiology of FOG in PD. Besides motor symptoms, non-motor symptoms, such as cognitive, affective, and autonomic functions were also more severely implicated in freezers compared to non-freezers in our PD cohort. These findings are consistent with other longitudinal studies in other Countries (Ehgoetz Martens et al., 2018; Kim et al., 2018). Base on a PPMI cohort, presynaptic striatal dopaminergic depletion could predict the later development of FOG in *de novo* PD, patients with severe DAT uptake in striatum had a high incidence of FOG during follow-up (Kim et al., 2018). Besides, extra-striatal pathological conditions may also contribute to FOG. As shown in an *in-vivo* PET study, PD with freezers had more severe loss of striatal dopaminergic and cortical cholinergic binding, and the presence of cortical amyloidopathy, compared with non-freezers (Bohnen et al., 2014). These clinical and neuroimaging studies indicated that FOG is not merely a gait problem, but may represent a unique entity in PD with extensive neurodegeneration both in dopaminergic and non-dopaminergic system.

This is the first study in China to show that PD with FOG had increased latency of VGS. Previously, Nemanich et al. in 2016 from United States found that PD with FOG had prolonged saccade latency (Nemanich and Earhart, 2016) relative to PD without FOG and healthy controls. Ours is the second one worldwide to indicate such association. In addition, we also found that increased saccade latency was associated with a higher FOGQ score in PD, indicating saccade latency was fit for identification and progression of FOG in PD. It is well-known that frontal and parietal eye fields are the key regions for saccade generation. Saccade latency in VGS is negatively associated with gray matter volume in frontal and parietal eye fields and cerebellar vermis, as demonstrated in one saccade-neuroimaging study (Perneczky et al., 2011). Accumulating neuroimaging evidence indicated that PD with FOG had widespread structural and functional impairment in cortical and subcortical brain structures, especially the specific nodes of frontoparietal cortex, basal ganglia, mesencephalic, and cerebellar locomotor region (Bharti et al., 2019). Whereas, functional connectivity abnormality of cerebellar locomotor region was also critical to lesion-induced FOG patients, as disclosed in a lesion network mapping study (Fasano et al., 2017). Therefore, structural and functional impairment in frontoparietal cortex or cerebellar locomotor region could impact both oculomotor and FOG networks, which may explain the close association between prolonged saccade latency and FOG phenotype in PD. Multimodal MRI studies are warranted in future to verify this hypothesis.

Our result has important clinical implications. As VGS is relatively easy to perform and saccade latency is an objective oculomotor parameter, VNG may provide important information for detection and progression of FOG. There are some methodological limitations in the present study. As a cross-sectional study, only associated factor with FOG in PD could be summarized and we didn’t further stratify the FOG subtypes. Also, the sample size of FOG positive group in VNG study is limited. Studies with a larger sample size is warranted in future to verify the diagnostic value of saccade latency in FOG identification. Besides, wearable devices in combination with FOGQ could be more objective to investigate the close relationship between gait parameters and oculomotor dynamics (Iijima et al., 2017).

## Conclusion

In summary, our results verify that FOG is a unique entity in PD with more severe motor and non-motor symptoms. Prolonged saccade latency could be a practical oculomotor parameter both for identification and progression of FOG in PD.

## Data Availability Statement

The raw data supporting the conclusions of this article will be made available by the authors, without undue reservation.

## Ethics Statement

The studies involving human participants were reviewed and approved by the Ethics Committee of Shanghai Ninth People’s Hospital, Shanghai Jiao Tong University School of Medicine. The patients/participants provided their written informed consent to participate in this study.

## Author Contributions

LW contributed to conception, organization, and execution of research project, design, execution, and review and critique of statistical analysis, and writing of the first draft of the manuscript. QW contributed to conception of research project, design, execution, and review and critique of statistical analysis, and writing of the first draft of the manuscript. LZ contributed to organization and execution of research project, design, execution, and review and critique of statistical analysis, and writing of the first draft of the manuscript. C-YJ and QX contributed to organization and execution of research project. S-CW contributed to execution and review and critique of statistical analysis. Y-RD contributed to organization and execution of research project and review and critique of the manuscript. QH contributed to organization of research project, design and execution of statistical analysis, and writing of the first draft and review and critique of the manuscript. WC contributed to conception and organization of research project, execution and review and critique of statistical analysis, and writing of the first draft and review and critique of the manuscript. J-RL contributed to conception of research project, review and critique of statistical analysis, and review and critique of the manuscript. All authors contributed to the article and approved the submitted version.

## Conflict of Interest

The authors declare that the research was conducted in the absence of any commercial or financial relationships that could be construed as a potential conflict of interest.

## References

[B1] Amboni M. Stocchi F. Abbruzzese G. Morgante L. Onofrj M. Ruggieri S. (2015). Prevalence and associated features of self-reported freezing of gait in Parkinson disease: the DEEP FOG study. *Parkinsonism Relat. Disord.* 21 644–649. 10.1016/j.parkreldis.2015.03.028 25899545

[B2] Anderson T. J. MacAskill M. R. (2013). Eye movements in patients with neurodegenerative disorders. *Nat. Rev. Neurol.* 9 74–85. 10.1038/nrneurol.2012.273 23338283

[B3] Bharti K. Suppa A. Tommasin S. Zampogna A. Pietracupa S. Berardelli A. (2019). Neuroimaging advances in Parkinson’s disease with freezing of gait: a systematic review. *NeuroImage Clin.* 24:102059. 10.1016/j.nicl.2019.102059 31795038 PMC6864177

[B4] Bohnen N. I. Frey K. A. Studenski S. Kotagal V. Koeppe R. A. Constantine G. M. (2014). Extra-nigral pathological conditions are common in Parkinson’s disease with freezing of gait: an in vivo positron emission tomography study. *Mov. Disord.* 29 1118–1124. 10.1002/mds.25929 24909584 PMC4162130

[B5] Chen K.-L. Xu Y. Chu A.-Q. Ding D. Liang X.-N. Nasreddine Z. S. (2016). Validation of the chinese version of montreal cognitive assessment basic for screening mild cognitive impairment. *J. Am. Geriatr. Soc.* 64 e285–e290. 10.1111/jgs.14530 27996103

[B6] Chen W. Chen S. Kang W.-Y. Li B. Xu Z.-M. Xiao Q. (2012). Application of odor identification test in Parkinson’s disease in China: a matched case-control study. *J. Neurol. Sci.* 316 47–50. 10.1016/j.jns.2012.01.033 22364958

[B7] Ehgoetz Martens K. A. Lukasik E. L. Georgiades M. J. Gilat M. Hall J. M. Walton C. C. (2018). Predicting the onset of freezing of gait: a longitudinal study. *Mov. Disord.* 33 128–135. 10.1002/mds.27208 29150872

[B8] Ewenczyk C. Mesmoudi S. Gallea C. Welter M.-L. Gaymard B. Demain A. (2017). Antisaccades in Parkinson disease: a new marker of postural control? *Neurology* 88 853–861. 10.1212/WNL.0000000000003658 28130466

[B9] Fasano A. Laganiere S. E. Lam S. Fox M. D. (2017). Lesions causing freezing of gait localize to a cerebellar functional network. *Ann. Neurol.* 81 129–141. 10.1002/ana.24845 28009063 PMC5266642

[B10] Giladi N. McDermott M. P. Fahn S. Przedborski S. Jankovic J. Stern M. (2001). Freezing of gait in PD: prospective assessment in the DATATOP cohort. *Neurology* 56 1712–1721. 10.1212/wnl.56.12.1712 11425939

[B11] Giladi N. Shabtai H. Simon E. Biran S. Tal J. Korczyn A. (2000). Construction of freezing of gait questionnaire for patients with Parkinsonism. *Parkinsonism Relat. Disord.* 6 165–170. 10.1016/s1353-8020(99)00062-0 10817956

[B12] Hamilton M. (1960). A rating scale for depression. *J. Neurol. Neurosurg. Psychiatry* 23 56–62. 10.1136/jnnp.23.1.56 14399272 PMC495331

[B13] Hoehn M. M. Yahr M. D. (1967). Parkinsonism: onset, progression and mortality. *Neurology* 17 427–442. 10.1212/wnl.17.5.427 6067254

[B14] Iijima M. Mitoma H. Uchiyama S. Kitagawa K. (2017). Long-term monitoring gait analysis using a wearable device in daily lives of patients with parkinson’s disease: the efficacy of selegiline hydrochloride for gait disturbance. *Front. Neurol.* 8:542. 10.3389/fneur.2017.00542 29114238 PMC5660685

[B15] Kang G. A. Bronstein J. M. Masterman D. L. Redelings M. Crum J. A. Ritz B. (2005). Clinical characteristics in early Parkinson’s disease in a central California population-based study. *Mov. Disord.* 20 1133–1142. 10.1002/mds.20513 15954133 PMC3643967

[B16] Katzman R. Zhang M. Y. Ouang Y.-Q. Wang Z. Y. Liu W. T. Yu E. (1988). A Chinese version of the mini-mental state examination; impact of illiteracy in a Shanghai dementia survey. *J. Clin. Epidemiol.* 41 971–978. 10.1016/0895-4356(88)90034-0 3193141

[B17] Kim R. Lee J. Kim Y. Kim A. Jang M. Kim H.-J. (2018). Presynaptic striatal dopaminergic depletion predicts the later development of freezing of gait in de novo Parkinson’s disease: an analysis of the PPMI cohort. *Parkinsonism Relat. Disord.* 51 49–54. 10.1016/j.parkreldis.2018.02.047 29523394

[B18] Nemanich S. T. Earhart G. M. (2016). Freezing of gait is associated with increased saccade latency and variability in Parkinson’s disease. *Clin. Neurophysiol.* 127 2394–2401. 10.1016/j.clinph.2016.03.017 27178858 PMC4867191

[B19] Nieuwboer A. Giladi N. (2013). Characterizing freezing of gait in Parkinson’s disease: models of an episodic phenomenon. *Mov. Disord.* 28 1509–1519. 10.1002/mds.25683 24132839

[B20] Nomura T. Inoue Y. Kagimura T. Uemura Y. Nakashima K. (2011). Utility of the REM sleep behavior disorder screening questionnaire (RBDSQ) in Parkinson’s disease patients. *Sleep Med.* 12 711–713. 10.1016/j.sleep.2011.01.015 21700495

[B21] Perez-Lloret S. Negre-Pages L. Damier P. Delval A. Derkinderen P. Destée A. (2014). Prevalence, determinants, and effect on quality of life of freezing of gait in Parkinson disease. *JAMA Neurol.* 71 884–890. 10.1001/jamaneurol.2014.753 24839938

[B22] Perneczky R. Ghosh B. C. P. Hughes L. Carpenter R. H. S. Barker R. A. Rowe J. B. (2011). Saccadic latency in Parkinson’s disease correlates with executive function and brain atrophy, but not motor severity. *Neurobiol. Dis.* 43 79–85. 10.1016/j.nbd.2011.01.032 21310235 PMC3102178

[B23] Postuma R. B. Berg D. Stern M. Poewe W. Olanow C. W. Oertel W. (2015). MDS clinical diagnostic criteria for Parkinson’s disease. *Mov. Disord.* 30 1591–1601. 10.1002/mds.26424 26474316

[B24] Richards M. Marder K. Cote L. Mayeux R. (1994). Interrater reliability of the Unified Parkinson’s Disease Rating Scale motor examination. *Mov. Disord.* 9 89–91. 10.1002/mds.870090114 8139610

[B25] Schlenstedt C. Muthuraman M. Witt K. Weisser B. Fasano A. Deuschl G. (2016). Postural control and freezing of gait in Parkinson’s disease. *Parkinsonism Relat. Disord.* 24 107–112. 10.1016/j.parkreldis.2015.12.011 26762797

[B26] Tomlinson C. L. Stowe R. Patel S. Rick C. Gray R. Clarke C. E. (2010). Systematic review of levodopa dose equivalency reporting in Parkinson’s disease. *Mov. Disord.* 25 2649–2653. 10.1002/mds.23429 21069833

[B27] Verbaan D. Marinus J. Visser M. van Rooden S. M. Stiggelbout A. M. van Hilten J. J. (2007). Patient-reported autonomic symptoms in Parkinson disease. *Neurology* 69 333–341. 10.1212/01.wnl.0000266593.50534.e8 17646625

[B28] White O. B. Saint-Cyr J. A. Tomlinson R. D. Sharpe J. A. (1983). Ocular motor deficits in Parkinson’s disease. II. Control of the saccadic and smooth pursuit systems. *Brain* 106(Pt 3), 571–587. 10.1093/brain/106.3.571 6640270

