# Supplementary material for: Clinical and Oculomotor Correlates With Freezing of Gait in a Chinese Cohort of Parkinson’s Disease Patients
Source: Front Aging Neurosci. 2020 Jul 31;12:237. doi: 10.3389/fnagi.2020.00237 (PMC7438737; doi:10.3389/fnagi.2020.00237)
Supplement: Supplementary file 1 [file Table_1.DOCX]

**Clinical and oculomotor correlates with freezing of gait**

**in a Chinese cohort of Parkinson’s disease patients**

**Supplementary material**

Li Wu^1†^, Qin Wang^2†^, Lei Zhao^1†^, Chun-Yan Jiang^1^, Qian Xu^1^, Si-Cheng Wu^3^, You-Rong Dong^1^, Qing He^1*^, Wei Chen^1*^, Jian-Ren Liu^1^

^1^ *Department of Neurology, Shanghai Ninth People’s Hospital, Shanghai Jiao Tong University School of Medicine, 200011, Shanghai, China*

^2^ *Department of Neurology, Zhongshan Hospital Fudan University, Shanghai 200032, China*

^3^ *Biostatistics Office of Clinical Research Center, Shanghai Ninth People’s Hospital, Shanghai Jiao Tong University School of Medicine, 200011, Shanghai, China*

**Supplementary Table 1 Demographic and clinical data among three groups with videonystagmography**

|  | **PD with FOG** | **PD without FOG** | **Healthy controls** | **P value** |
| --- | --- | --- | --- | --- |
| Number | 20 | 20 | 37 |  |
| Age, y | 67.0±6.0 | 65.6±3.9 | 63.6±7.5 | 0.138 |
| M/F, *n* | 11/9 | 12/8 | 24/13 | 0.762 |
| Disease duration, y | 5.0(1.2, 6.7) | 4.0(2.0, 6.7) | na | 0.844 |
| Hoehn and Yahr stage |  |  | na | 0.507 |
| 1-2, *n*(%) | 12(60.0) | 14(70.0) |  |  |
| >2, *n*(%) | 8(40.0) | 6(30.0) |  |  |
| UPDRS-II (0-52) | 15.9±5.9 | 10.0±3.8 | na | 0.001 |
| UPDRS-III (0-108) | 32.4±14.4 | 32.0±11.5 | na | 0.558 |
| Wearing-off, *n*(%) | 9(45) | 8(40) | na | 0.746 |
| NMSQuest (0-30) | 10.3±4.2 | 9.0±4.3 | - | 0.358 |
| SS-16 (0-16) | 7.4±3.0 | 7.5±3.0 | - | 0.875 |
| RBDSQ (0-13) | 4.0(1.5, 6.7) | 3.5(1.0, 7.0) | - | 0.915 |
| HAMD-17 (0-17) | 7(3.2, 12.0) | 6(3.2, 10.0) | - | 0.528 |
| SCOPA-AUT (0-63) | 15.5(12.2, 18.0) | 11.5(9.0, 17.2) | - | 0.091 |
| MMSE (0-30) | 27.7±2.0 | 27.5±2.1 | 28.0±1.6 | 0.819 |
| MoCA-BC (0-30) | 21.4±4.4 | 21.4±2.8 | - | 0.932 |
| LED, mg/d | 425.0(250.0, 650.0) | 406.2(290.6,593.7) | na | 0.647 |

Notes: FOG, freezing of gait; M/F, male/female; UPDRS, Unified Parkinson’s Disease Rating Scale ; NMSQuest, Non-motor Symptoms Questionnaire; SS-16, 16-item odor identification test from Sniffin’ Sticks; RBDSQ, REM Behavior Disorder Screening Questionnaire; HAMD-17, 17-item Hamilton Rating Scale for Depression; SCOPA-AUT, The Scale for Outcomes in PD autonomic dysfunction; MMSE, Mini Mental State Examination; MoCA-BC, [Montreal Cognitive Assessment Basic](https://www.ncbi.nlm.nih.gov/pubmed/29183843); LED, L-dopa equivalent dosage. na, not applicable; -, not done.
